# Membrane Sweeping to Induce Labor in Post-term Pregnant Women: Success Rate and Outcomes

**DOI:** 10.7759/cureus.36942

**Published:** 2023-03-31

**Authors:** Albagir M Hassan

**Affiliations:** 1 Obstetrics and Gynaecology, Umm Al-Qura University, Makkah, SAU

**Keywords:** post-dates, neonatal outcome, maternal outcome, induction of labour, sweeping of membranes

## Abstract

Introduction

Membrane sweeping is a mechanical technique by which a clinician inserts one or two fingers into the cervix and detaches the inferior pole of the membranes from the lower uterine segment using a continuous circular sweeping motion. This produces hormones that promote effacement and dilatation, potentially promoting labor. This study aimed to determine the success rate and the outcome of membrane sweeping in postdate pregnant women in Alhasahesa Teaching Hospital.

Methods

This prospective descriptive cross-sectional study conducted at Alhashesa Teaching Hospital, Alhashesa, Sudan, between May and October 2022 included all pregnant women at 40 or more weeks of gestation who underwent membrane sweeping to induce labor. We recorded the number of sweeps needed, sweeping-to-delivery interval, mode of delivery, maternal outcome, and fetal outcome (including birth weight, Apgar score at delivery, and the need for neonatal intensive care unit admission [NICU]). Data were collected through patient interviews using a specially designed questionnaire and analyzed using Statistical Package for Social Sciences (SPSS®) software for Windows, Version 26.0 (Armonk, NY: IBM Corp.),

Results

Membrane sweeping induced labor in 127 postdate women (86.4%). Most of the women in the study (n=138; 93.9%) had no complications, seven (4.8%) had postpartum hemorrhage, one (0.7%) had sepsis, and one (0.7%) was admitted to the intensive care unit. All neonates were alive, and most (n=126; 85.8%) birth weights ranged from 2.5 kg to 3.5 kg. Thirteen (8.8%) neonates weighed less than 2.5 kg, and eight (5.4%) weighed more than 3.5 kg. One hundred thirty-three (90.5%) had Apgar scores <7, eight (5.4%) had Apgar scores under five, and six (4.1%) had Apgar scores of five to six. Seven neonates (4.8%) were admitted to the NICU.

Conclusions

Membrane sweeping to induce labor has a high success rate, and it can be safe for both the mother and the baby, as it is associated with a low rate of maternal and fetal complications. Additionally, no maternal and/or fetal deaths were reported. A large, controlled study is required to compare its benefits over other methods of induction of labor.

## Introduction

A pregnancy that continues beyond 42 weeks gestation is a post-term pregnancy, which poses significant risks to both the mother and her child [1]. Fetal growth beyond this point is associated with reduced placental reserve and blood supply, leading to increased fetal and neonatal morbidity and mortality [2]. While the mean length of a typical pregnancy is around 40 weeks, calculated from the first day of the last menstrual period, estimating gestational age using first-trimester ultrasound and measuring the crown-rump length (CRL) or head circumference when the CRL is greater than 84 mm is more accurate [3].

Risk factors for post-term pregnancy include being a first-time mother, having had a previous post-term pregnancy, and congenital fetal malformations (such as anencephaly) [4]. Post-term pregnancy is associated with fetal complications such as oligohydramnios, intrapartum fetal asphyxia [4], meconium aspiration, stillbirth, and trauma during labor (such as shoulder dystocia) [5]. Maternal complications can include prolonged and obstructed labor, increased risk of operative vaginal delivery and cesarean section, genital tract trauma and hemorrhage, and heightened maternal anxiety [5].

Inducing labor is a recognized intervention to improve outcomes and reduce complications related to post-term pregnancy, but there are ongoing debates regarding the need and timing of labor induction. This depends on various factors, including the accuracy of gestational age calculation, previous obstetric history, estimated fetal weight, cervical score, and maternal preference [6]. Various methods for labor induction exist, including prostaglandins, which play a key role in cervical ripening and initiating uterine contractions in both ripened and un-ripened cervixes. Oxytocin infusion is associated with initiating rhythmic uterine contractions and is usually used in patients with a higher cervical score. Both medical drugs used for labor induction have adverse effects, such as uterine hyperstimulation and intrapartum fetal distress, especially when combined with other mechanical methods for induction [7]. Mechanical labor induction methods include osmotic dilators, transcervical Foley catheters, double-balloon catheters, and membrane sweeping (stripping) [8].

Membrane sweeping is performed during a vaginal exam, during which the examiner inserts one or two fingers inside the cervical canal and rotates them in a circular motion to separate the lower part of the amniotic membranes from the lower uterine wall. This process releases endogenous prostaglandins, leading to cervical ripening and initiation of uterine contractions. Adverse effects of these methods include vaginal bleeding after examination and maternal discomfort [9]. This study aimed to determine the success rate and outcome of membrane sweeping in post-term pregnant women at Alhashesa Teaching Hospital, Alhashesa, Sudan, from May to October 2022. Additionally, this study aimed to determine fetal outcomes associated with membrane sweeping.

## Materials and methods

Study design

We conducted a prospective descriptive cross-sectional study at Alhashesa Teaching Hospital between May and October 2022. 147 women were included in the study. We consider 40 weeks or more of gestation as the threshold to begin membrane sweeping to achieve vaginal delivery, so we included all pregnant women at 40 or more weeks of gestation in this study. We excluded women who declined to participate, pregnant women with intrauterine fetal demise, and women with any previous medical conditions and obstetrical complications. All patients underwent membrane sweeping to induce labor, repeated every 72 hours if no response, the maximum number of sweeping needed was four times. No added methods of induction were used once uterine contractions started and labor was managed as labor progressed. We measured the response by recording the number of sweeps needed, sweeping-to-delivery interval, mode of delivery, maternal outcome, and fetal outcome (including birth weight, Apgar score at delivery, and the need for neonatal intensive care unit [NICU] admission). We collected data by conducting patient interviews using a specially designed questionnaire that included questions related to the study objectives. The questionnaire also gathered basic patient information, including obstetric history, the outcome of membrane sweeping, maternal outcome, and fetal outcome.

Data analysis

We analyzed the data using Statistical Package for Social Sciences (SPSS®) software for Windows, version 26.0 (IBM Corp., Armonk, USA). Numerical variables were presented as mean and standard deviation. For categorical variables, frequency and percentages were used.

Ethical consideration

Ethical approval was taken from the Ethics Committee at the Research Unit and provided to the hospital administration (Alhashesa Teaching Hospital issued approval 1264). We obtained written consent from participants after explaining the nature and purpose of the study. Confidentiality of the data collected was considered by not involving the names and identifications and using the information solely for research purposes.

## Results

This prospective descriptive cross-sectional study enrolled 147 pregnant women at 40 weeks of gestational age or more at Alhashesa Teaching Hospital from May to October 2022. Table 1 presents the demographic characteristics of the study population, the mean age 27.97 ± 7.801 years with 25 patients (17%) under the age of 20 years, 50 (34%) aged 20 to 29 years, 55 (37.4%) aged 30 to 39 years, and 17 (11.6%) older than 39 years. Thirty-one (21.1%) patients were illiterate, 25 (17%) had primary education, 79 (53.7%) had secondary education, and 12 (8.2%) had a university education.

**Table 1:** Distribution of patients with membrane sweeping according to patient demographics.

Demographic	N (%)
Age in years	(Mean age 27.97±7.801 years)
< 20 years	25 (17%)
20-29 years	50 (34%)
30-39 years	55 (37.4%)
> 39 years	17 (11.6%)
Total	147 (100%)
Level of education	
Illiterate	31 (21.1%)
Primary education	25 (17%)
Secondary education	79 (53.7%)
University education	12 (8.2%)
Total	147 (100%)

Of the 147 patients, 54.4% were gravida two to four, 25.9% were primigravida, and 19.7% were gravida five or more. Regarding antenatal care, 26 (17.7%) patients had no antenatal visits, 40 (27.2%) had irregular visits, and 81 (55.1%) had regular visits. The mode of previous delivery, with 45 patients (30.6%) having no previous delivery, 69 (46.9%) having a vaginal delivery, six (4.1%) having an instrumental vaginal delivery, and 27 (18.4%) having a cesarean section (Table 2).

**Table 2:** Distribution of patients with membrane sweeping according to obstetric characteristics

Antenatal Care	N (%)
No antenatal care	26 (17.7%)
Irregular antenatal care	40 (27.2%)
Regular antenatal care	81 (55.1%)
Total	147 (100%)
Gravidity	
Primigravida	38 (25.9%)
2-4	80 (54.4%)
5 and more	29 (19.7%)
Total	147 (100%)
Mode of previous delivery	
Not delivered before	45 (30.6%)
Vaginal delivery	69 (46.9%)
Instrumental vaginal delivery	6 (4.1%)
Caesarean section	27 (18.4%)

Table 3 shows that 100 (68%) patients had no previous history of post-term birth, while 47 (32%) had a history of post-term pregnancy.

**Table 3:** Distribution of patients with membrane sweeping according to history of post-term pregnancy

History of Previous Post-term	N (%)
No	100 (68%)
Yes	47 (32%)
Total	147 (100%)

Table 4 shows the gestational age at presentation, with 42 (28.6%) patients presenting at 40 to 41 weeks, 65 (44.2%) at 41 to 42 weeks, and 40 (27.2%) beyond 42 weeks.

**Table 4:** Distribution of patients with membrane sweeping according to gestational age at the time of presentation

Gestational Age at Presentation	N (%)
40-41 weeks	42 (28.6%)
41-42 weeks	65 (44.2%)
More than 42 weeks	40(27.2%)
Total	147 (100%)

Of the 147 patients who underwent membrane sweeping, 57 (38.8%) required only one trial, 70 (47.6%) had two trials, and 20 (13.6%) had more than two trials, mean (1.634±0.078 trials) (Table 5).

The mean interval between sweeping and delivery was 36.55±42.04 hours; the interval between sweeping and delivery was less than 24 hours in 61 (41.5%), between 24 hours and one week in 79 (53.7%), and more than one week in seven (4.8%) patients (Table 6).

**Table 5:** Distribution of patients with membrane sweeping according to interval between sweeping and labor

Interval Between Membrane Sweeping to Labor	N (%)
(Mean 36.55±42.04 hours;)	
Less than 24 hours	61 (41.5%)
24 hours to 1 week	79 (53.7%)
More than 1 week	7 (4.8%)
Total	147 (100%)

Regarding delivery mode, vaginal delivery was achieved in 127 (25 primigravidas and 102 multigravidas) (86.4%) patients, instrumental vaginal delivery in four (three primigravidas and one multigravida) (2.7%), and cesarean section in 16 (10 primigravidas and six multi gravidae) (10.9%;) (Table 7).

**Table 6:** Distribution of patients with membrane sweeping according to mode of delivery

Mode of Delivery After Sweeping	Primigravida N (%)	Multigravida N (%)	Total N (%)
Vaginal delivery	25 (17%)	102 (69.4%)	127 (86.4%)
Instrumental vaginal delivery	3 (2.04%)	1 (0.66 %)	4 (2.7%)
Caesarean section	10 (6.80%)	6 (4.1 %)	16 (10.9%)
Total	38 (25.85 %)	109 (74.15 %)	147 (100%)

Maternal outcomes are shown in Table 8, with 138 (93.9%) patients experiencing no complications, seven (4.8%) had postpartum hemorrhage, and two (1.3%) had sepsis. All patients were discharged from the hospital in good health.

**Table 7:** Distribution of patients with membrane sweeping according to maternal outcome

Maternal outcome	N (%)
No complications	138 (93.9%)
Postpartum hemorrhage	7 (4.8%)
Sepsis	2 (1.3%)
Total	147 (100%)

Neonatal outcomes are presented in Table 9, with all babies born alive. Thirteen (8.8%) had a birth weight less than 2.5 kg, 126 (85.8%) had a birth weight between 2.5 to 3.5 kg, and eight (5.4%) had a birth weight greater than 3.5 kg. Regarding the Apgar score at delivery, 133 (90.5%) had a score ≥7, six (4.1%) had a score between 5 to 6, and eight (5.4%) had a score <5. Seven (4.8%) babies required admission to the NICU, and all were discharged from the hospital in good health.

**Table 8:** Distribution of patients with membrane sweeping according to neonatal outcome

Neonatal Outcome	N (%)
Alive	147 (100%)
Stillbirth	0 (0%)
Total	147 (100%)
Birth weight	
<2.5 kg	13 (8.8%)
2.5–3.5 kg	126 (85.8%)
>3.5	8 (5.4%)
Total	147 (100%)
Apgar score at delivery	
<5	8 (5.4%)
5-6	6 (4.1%)
7 and more	133 (90.5%)
Total	147 (100%)
NICU admission	
No admission needed	140 (95.2%)
Admitted to NICU	7 (4.8%)
Total	147 (100%)

## Discussion

There were many arguments about the effect and outcome of starting membrane sweeping before the gestational age of 42 weeks, Yildirim et al, a randomized controlled trial (RCT), compared the effects of membrane sweeping at 38 to 40 weeks gestation (including 179 participants) to only performing pelvic examination (including 167 participants). They concluded that the average delivery time was 4 days in the sweeping group compared to eight days in the control group (p<0.0001) [10]. Similarly, de Miranda et al compared starting membrane sweeping - at 41 weeks gestational age - with expectant management in preventing post-date pregnancy including 742 low-risk participants. Serial sweeping of the membranes at 41 weeks gestation decreased the risk of post-term pregnancy without more significant adverse neoanatal outcomes [11]. In this study we used 40 weeks of gestation to start membrane sweeping aiming to achieve delivery. Also, this study aimed to evaluate the effectiveness and safety of membrane sweeping for labor induction in women with post-term pregnancy. Our results indicate that membrane sweeping had a success rate of 86.4% in achieving vaginal delivery, consistent with previous studies [12]. Most patients (86.4%) required only one or two sweeps to initiate labor, while a minority (13.6%) needed more than two sweeps. No other methods were used as induction, which is comparable to the results of Lella et al [13]. These findings suggest that membrane sweeping is a practical and efficient method for labor induction in postdate pregnancies.

Regarding maternal outcomes, most (93.9%) of the patients had no complications, 4.8% experienced postpartum hemorrhage, and 1.3% had sepsis. These rates are similar to those reported by Ali et al., who found no significant side effects of membrane sweeping [14]. The procedure was generally well-tolerated, with most women experiencing only discomfort during the vaginal exam.

All fetuses in our study were born alive, with a majority (90.5%) with an Apgar score ≥7. A small proportion (5.4%) had an Apgar score <5, and 4.8% required admission to the NICU. These rates are similar to those reported by Nyamzi et al. in Nigeria, which evaluated the effectiveness of sweeping of membranes to reduce the incidence of elective induction of labor and to compare pregnancy outcomes in women who had sweeping of membranes after 40 weeks of gestation, which sowed that neonates with Apgar score > 7 were (3.1%) and (4.1%) required NICU admission [15]. These results suggest membrane sweeping is safe for both the mother and fetus in post-term pregnancies.

Overall, membrane sweeping showed good results in inducing labour in women with post-term pregnancy without adding significant risks for the mothers or the neonates. A meta-analysis study done by Avdiyovski et al revealed that membrane sweeping is effective in promoting spontaneous labour and reducing formal induction in post-term pregnancy, [16].

Limitations

Our study had several limitations. The study was conducted in a single hospital, which may limit the representations of the sample. The study did not include a control group, which may limit the ability to draw causal conclusions about the effectiveness of membrane sweeping. Finally, the study relied on patients’ self-report for some variables, which may introduce bias in the data.

## Conclusions

Based on the findings of this study, membrane sweeping appears to be a safe and effective method for labor induction in post-term pregnancies. The high success rate of achieving vaginal delivery and low rate of maternal and fetal complications make it an attractive option for clinical practice, particularly in low-resource settings. The absence of maternal and fetal deaths further supports the safety of this procedure. However, further studies are needed to determine the optimal timing and frequency of membrane sweeping in post-term pregnancies and its effectiveness in high-risk populations.

To maximize the benefits of membrane sweeping for labor induction, hospitals should establish a protocol for the induction of labor and provide appropriate facilities for fetal monitoring and neonatal care. To determine the probability of a successful outcome, healthcare providers should evaluate the cervix using the Bishop score and select the most appropriate induction method. Women should also be advised to closely follow up with antenatal care, particularly in the third trimester. By implementing these recommendations, membrane sweeping can provide a safe and effective alternative to medical or mechanical labor induction methods in post-term pregnancies.
